# OLDER ADULTS' PERSPECTIVES OF INDEPENDENCE THROUGH TIME: RESULTS OF A LONGITUDINAL INTERVIEW STUDY

**DOI:** 10.1093/geroni/igac059.611

**Published:** 2022-12-20

**Authors:** Emily Taylor, Julia Frost, Victoria Goodwin, Susan Ball, Andrew Clegg

**Affiliations:** University of Exeter, Truro, England, United Kingdom; University of Exeter, Exeter, England, United Kingdom; University of Exeter, Exeter, England, United Kingdom; University of Exeter, Exeter, England, United Kingdom; University of Leeds, Leeds, England, United Kingdom

## Abstract

Understanding how older people experience, and adapt to maintain, independence through time has implications for person-centred care. Quantifiable measures can provide a gauge of change in practice. However, little is known about how older people themselves perceive independence through time, or whether measures used are commensurate with what matters to older people. This study aimed to identify whether and how older adults assimilate their perceptions of independence in response to change through time. Two semi-structured interviews were conducted longitudinally, one year apart, to explore the views of 12 community-dwelling older adults, aged 76-85 years. A constructionist approach using dramaturgical and descriptive codes, facilitated the data interpretation. Sixteen analytical questions guided exploration of participants’ perceptions of independence through time. Interview participants felt that common interpretations of independence underestimated, and omitted, important aspects of their experience through time. Some participants questioned the value of instruments that were insensitive to individual values and context. Changes in life trajectories required participants to adapt the form, or means of obtaining independence. The impact of change on participants’ sense of independence was value-dependent, informed by the function a participant ascribed to maintaining independence. This study builds on the understanding of independence as a complex and multifaceted construct. The findings challenge the congruence of common interpretations of independence with older people’s views, showing areas of commonality and discrepancy. Exploration of independence in terms of form and function provides important understanding about how continuity of function takes precedence to form in determining the maintenance of independence through time.

